# Novel pathophysiological insights into CAR-T cell associated neurotoxicity

**DOI:** 10.3389/fneur.2023.1108297

**Published:** 2023-03-08

**Authors:** Vassilis Genoud, Denis Migliorini

**Affiliations:** ^1^Department of Oncology, University Hospital of Geneva, Geneva, Switzerland; ^2^Center for Translational Research in Onco-Haematology, University of Geneva, Geneva, Switzerland; ^3^Brain Tumor and Immune Cell Engineering Laboratory, AGORA Cancer Research Center, Lausanne, Switzerland; ^4^Swiss Cancer Center Léman (SCCL), Lausanne and Geneva, Geneva, Switzerland

**Keywords:** immune effector cell-associated neurotoxicity syndrome (ICANS), neurotoxicity, cellular therapies, chimeric antigen receptor (CAR) T cells, cytokine release syndrome (CRS)

## Abstract

Chimeric antigen receptor (CAR) T cell therapy represents a scientific breakthrough in the treatment of advanced hematological malignancies. It relies on cell engineering to direct the powerful cytotoxic T-cell activity toward tumor cells. Nevertheless, these highly powerful cell therapies can trigger substantial toxicities such as cytokine release syndrome (CRS) and immune cell-associated neurological syndrome (ICANS). These potentially fatal side effects are now better understood and managed in the clinic but still require intensive patient follow-up and management. Some specific mechanisms seem associated with the development of ICANS, such as cytokine surge caused by activated CAR-T cells, off-tumor targeting of CD19, and vascular leak. Therapeutic tools are being developed aiming at obtaining better control of toxicity. In this review, we focus on the current understanding of ICANS, novel findings, and current gaps.

## 1. Introduction

Chimeric antigen receptor (CAR) T cells have shown high efficacy in multiple hematological indications and are now widely implemented in many centers (1). Nevertheless, the management of patients receiving CAR-T cell therapies is beyond the clinical management of high-grade hematological diseases. Not all centers can offer this novel therapy to their patients, mainly due to the frequent need for intensive care unit management and high-end infrastructure to deliver cell-therapy products.

### 1.1. CAR-T cells

CARs are fusion proteins combining an antibody-based recognition part and intracellular activation and co-stimulation domains. Through cell engineering, human T cells can express these CARs, thereby conferring specificity to a cell surface antigen (Ag) of interest without major histocompatibility complex (MHC) restriction. CAR-T cells can target any type of cell depending on the epitope selected. The recognizing part of the receptor is composed of a single-chain variable fragment (scFv) derived from the variable portion of an antibody (Ab). Currently, CD-28 and 4-1BB are the two co-stimulatory domains approved in the clinic.

### 1.2. Secondary effects

CAR-T cells are exceptionally active cellular therapies and have brought unprecedented success to previously untreatable diseases. However, their high activity drives systemic toxicities, either cytokine release syndrome (CRS) or immune cell-associated neurologic syndrome (ICANS), sometimes referred to as neurotoxicity. These toxicities were not identified in mouse models but rapidly forced clinicians to adapt their management. As both are associated, we will introduce both CRS and ICANS, but the scope of this review is to focus on ICANS.

#### 1.2.1. CRS

CRS is the most common CAR-T cell therapy-related toxicity, and it has been reported to develop in 30–100% of patients (2). It is characterized by clinical symptoms of hyperthermia and oxygenation or cardiovascular alterations. If promptly identified and well managed, it is most often fully reversible. Pathophysiological studies suggest that CRS results from pyroptotic cell death (3). As CAR-T cell cytotoxicity relies mainly on the release of granzyme B, it induces rapid activation of caspase 3 in target cells. This enzyme will cleave gasdermine E, which forms pores in the tumor cell membrane, leading to pyroptosis.

Consequently, gasdermine D in surrounding macrophages will be cleaved by caspase 1, leading to cytokine release by macrophages, inducing CRS (4). In the central nervous system (CNS), microglia cells have phagocytic functions and also express gasdermine D that can exacerbate neurotoxicity through pyroptosis (5). Because CAR-T cells release a high concentration of perforin or granzyme B compared with cytotoxic T lymphocytes, more immunogenic pyroptosis will be induced through the gasdermin pathway compared with the more common apoptosis pathway induced by cytotoxic T lymphocytes. Previous pre-clinical studies linked CRS severity to gasdermine cleavage (4). In other words, the CAR-T cell mechanism of toxicity indicates a more immunogenic cell death, leading to more potent activation of surrounding cells such as myeloid cells, amplifying the release of cytokines.

#### 1.2.2. ICANS

ICANS is less common than CRS as only half of patients will experience this syndrome (2). It usually develops after CRS initiation, potentially illustrating a causal link. As for CRS, ICANS is generally reversible even though rare cases of fatal ICANS have been reported (< 1% of cases) (6). CD19 CAR-T cells are the most incriminated in the development of ICANS, and counterintuitively, intrathecal or intratumoral infusion of CAR-T cells for patients with glioblastoma does not induce ICANS (7, 8). Many advances have been made in understanding ICANS development, with multiple pre-clinical models and consensual grading of ICANS in patients (9). Since the pathophysiology is still poorly understood, we will address the most recent developments.

## 2. ICANS

### 2.1. Clinical presentation

ICANS may present with different symptoms, such as dysgraphia (6, 10), frontal lobe dysfunction (11), language disorders, or akinetic-mutism (9), and can rarely evolve into a seizure or fulminant cerebral edema (12, 13). It usually develops 4–6 days after CAR-T cell infusion and lasts 5–13 days (14, 15). Symptoms are fully reversible, but sometimes more prolonged toxicity can be observed (11, 16). Clinical workup includes laboratory measures, magnetic resonance imaging (MRI) studies, and electroencephalography (EEG). CAR-T cell kinetics of amplification and serum cytokine levels correlate with ICANS development (17, 18). Analysis of cerebrospinal fluid (CSF) is performed only in clinical trials and will be discussed later. MRI findings are most often unspecific (19), but studies described a potential specific pattern of edema in patients suffering from severe ICANS, primarily located in the bilateral thalami, supratentorial white matter, and brainstem region (10, 20). Abnormal EEG patterns can predict the development of clinical seizures in patients with ICANS (13, 21) and correlate with ICANS severity (22).

As ICANS was not anticipated from pre-clinical trials, it took some time to develop consensual grading. However, the American Society for Blood and Marrow Transplantation proposed a consensual definition and grading system (9) based on the clinical immune effector cell-associated encephalopathy score (6).

### 2.2. Pathophysiology

Better grading and reporting of ICANS in the clinic and the development of pre-clinical models of ICANS helped to decipher the mechanisms driving this syndrome. In particular, a CD19+ lymphoma xenograft model (23) and a humanized NSG mouse model (24) helped to improve our understanding. Nevertheless, they do not represent human cytokines and human hematopoietic cells and present xenograft vs. host reaction (25–28), thus limiting their direct translation.

Cytokines such as IL-6, IL-1, and TNF-α have been widely identified as at the root of CRS or ICANS, and their blockade in the clinic can limit CAR-T cell toxicities, as we will show later. Many other cytokines and cell subtypes are involved, and we will address them in the following sections.

#### 2.2.1. Cytokines

During clinical studies, thorough analyses of serum and CSF identified IL1-6, IL-1, IFN-γ, TNF-α, and GM-CSF (20) as major contributors to the overactivation of the peripheral immune system.

IL-6, identified as the critical regulator of CRS in many clinical trials, harbors pro- and anti-inflammatory effects and is mainly produced by the myeloid lineage. It has an autocrine activity to promote macrophage maturation and activation, and its receptor is also widely expressed on immune cells and controls the acute phase of inflammation (18). IL-6 has been described as responsible for fatal CRS in pre-clinical models (23, 25) and for promoting macrophage activation (23). In patients, tocilizumab, a monoclonal IL-6 receptor (IL-6R) blocking Ab, can limit most CRS symptoms and reverse cytokine levels (29, 30).

Nevertheless, IL-6R blockade has no impact on limiting ICANS, contrasting with the blocking of the IL-1 axis (23), hinting at the primary role of IL-1 in ICANS physiopathology. Following CAR-T cell infusion, IL-1 elevates before IL-6 in the serum (25, 31). IL-1 further induces monocyte activation and neutrophil infiltration into the brain (31). IL-1 is also highly implicated in CRS, and the use of anakinra, an IL-1 receptor blocker, in a mouse model showed reduced symptoms and mortality, while not affecting CAR-T cell activity (25).

IFN-γ is secreted by activated CAR-T cells and macrophages and exerts fundamental anti-tumor and pro-inflammatory activity (32). CAR-T cells depend highly on IFN-γ, and its blocking will decrease CAR-T function.

TNF-α is another cytokine identified during CAR-T cell toxicity. It activates myeloid cell proliferation, migration, and production of cytokines (33). However, TNF-α also has a direct cytotoxic activity on target cells (34, 35). TNF-α blockade in the SCID-Beige model prevents IL-6 production by myeloid cells and limits CRS mortality, but also limits the efficacy of CAR-T cells (23).

GM-CSF essentially promotes differentiation and all effector functions of myeloid cells and has a central role in tissue inflammation (36, 37).

In conclusion, there is a clear link between serum cytokines and the development of constantly elevated ICANS in multiple studies with different constructs (13, 21, 30). Many cell types are implicated in producing these cytokines. However, a single-cell RNA sequencing study in mice identified that if many cell types produce IL-6, macrophages are the primary producer by far (23).

#### 2.2.2. Myeloid cells

Activated CAR-T cells release IFN-γ, TNF-α, and GM-CSF (38), which are cytotoxic on tumor cells but will also activate myeloid cells. Macrophages are also activated by damage-associated molecular patterns such as ATP, HMGB1, histone H3, and other signals through Toll-like receptors resulting from tumor and surrounding cell death, and macrophages will, in turn, further release IL-6 and TNF-α (37). Interestingly, macrophages must be activated by functional CAR-T cells, as in patients not responding to CAR-T cell therapy, there is no CRS induction (39).

As GM-CSF can lead to IL-6 production, its blocking can be protective against the development of CRS and neurotoxicity without compromising CAR-T cell efficacy. Moreover, CAR-T cell KO for GM-CSF showed better cytotoxic activity (40). Conversely, monocyte ablation negatively affects CAR-T proliferation and expansion (25).

#### 2.2.3. Endothelial cell activation and blood–brain barrier dysfunction

In the CNS, vascular exchanges with the parenchyma are highly controlled by endothelial cells (EC), which form with pericytes, smooth muscle cells, and astrocytes' end foots the blood–brain barrier (BBB) (41).

The EC permeability is regulated by the vascular endothelial growth factor (VEGF) (42) and angiopoietin (Ang)/tyrosine kinase with immunoglobulin-like and EGF-like domains (Tie) axis (43, 44) and is altered during CAR-T cell treatment (13). At constitutional levels, Ang1 is produced by platelets and perivascular cells and binds to Tie2 to stabilize the endothelium. Nevertheless, when activated by inflammatory cytokines, EC will release Ang2 and displace Ang1, leading to a vascular leak. Consequently, a higher Ang2/Ang1 ratio has been linked to higher ICANS severity (45), and Ang1 overexpression in mice preserved EC function and integrity (46). Adhesion molecules such as vascular cell adhesion protein 1 and intercellular adhesion molecule 1 are overexpressed during the high-inflammatory state caused by CAR-T cell treatment and facilitate leukocyte infiltration (33, 47).

Moreover, EC and exposed pericytes will produce IL-6 and VEGF in response to inflammation and, in particular excessive IFN-γ (48–51), affecting the BBB tight junctions and further worsening vascular leak (52, 53). In contact to excessive TNF-α, EC will also produce matrix metalloproteinase 2 and 9, further disrupting cell–matrix adhesion and contributing to increasing permeability (54), which can even lead to cerebral edema (55).

In a rhesus macaque pre-clinical model of ICANS, studying a CD20 CAR-T cell with 4-1BB co-stimulation domain, an increased concentration of cytokines was identified in the CSF. Moreover, a higher infiltration of CAR-T cells with increased expression of the integrin VLA4 was identified. Histological analysis revealed panencephalitis, with multifocal meningitis, and perivascular T-cell infiltration 8 days after infusion (56). In another model, IL-6R blockade did not ameliorate meningeal thickening and macrophage infiltration to the brain, but IL-1 blockade did (25). CAR-T cells with KO of GM-CSF led to decreased IL-6 levels and downregulated interactions with myeloid cells, leading to a restored endothelium permeability state (40, 57). In addition, in clinical trials, higher levels of CAR-T cells and cytokines in the CSF have been linked to higher-grade ICANS (21, 58, 59).

The thorough investigation of fatal brain edema cases during CAR-T cell therapy could identify different associated risk factors, such as younger age, higher CD8 ratio, higher IL-15 serum concentrations, and low platelets before infusion, as well as rapid expansion and higher IL-2 and TNF-α peak (60). Pathological examination of the brain revealed BBB disruption but no activated T cells in the CNS (60). In other words, many findings indicate the incriminating role of vascular leak due to BBB breakdown with elevated cytokine levels in the CNS. However, if CAR-T cells are also found in the CSF, it seems that they are not required for ICANS development.

#### 2.2.4. Other factors associated with ICANS

##### 2.2.4.1. Other soluble factors

Catecholamines are also elevated during CRS and ICANS (61). Adrenaline and noradrenaline have direct activation functions on CAR-T cells and will promote subsequent cytokine release (62). Therefore, by limiting this amplifying loop, CRS can be limited (61). Nevertheless, as severe CRS is defined by cardiovascular instability, limiting catecholamines could be detrimental.

Phosphorus has also been described to be associated with ICANS (63). The high metabolic activity during the CAR-T cell expansion phase may decrease phosphorus availability, and hypophosphatemia can lead to neurologic symptoms similar to ICANS. If a causality link is still to be proven, phosphorus supplementation would be easy to implement.

##### 2.2.4.2. Disseminated intravascular coagulation

Similarities with disseminated intravascular coagulation and sepsis are present in severe CRS or ICANS with hypofibrinogenemia and increased fibrin degradation, leading to endothelial cell disruption (18). Elevated serum D-Dimers decreased fibrinogen, and platelets seem to indicate a thrombotic microangiopathy process that compromises the BBB (13, 14). Activated and impaired vascular integrity can expose tissue factors, and collagen fibers triggering coagulation pathways has been observed (64).

##### 2.2.4.3. CNS infiltration of immune cells

Many studies identified CAR-T cells in the CSF of patients suffering from ICANS (48, 58, 65), with different phenotypes such as Th1, Th17, and regulatory T cells (25). Macrophages were also identified in the CNS in fatal CAR-T cell therapy cases, with parenchyma infiltration and expansion in the perivascular space (12, 66). A xenograft mouse model used GM-CSF neutralization to reduce CNS infiltration of CAR-T cells and myeloid (40), hinting at a potential therapeutic lead, even though there is no clear causality link between CAR-T cell CNS infiltration and ICANS development.

##### 2.2.4.4. Astrocytes and microglia activation

Glial fibrillary acidic protein (GFAP), a validated marker for astroglial cells injury (67), and S100b, a marker of astrocyte activation and injury (68), were both elevated in the CSF of patients experiencing ICANS (69). Activated astrocytes are also described in inflammatory or degenerative diseases such as multiple sclerosis, Alzheimer's disease, and Parkinson's disease (70). IFN-γ has a direct toxic effect on astrocytes, leading to further CNS inflammation and immune infiltration (47, 71). Astrocytes also interact with neurons by producing glutamate (54). When exposed to IL-1, astrocytes show a decreased functionality in glutamate signaling (55). Neuron activity is also affected by excessive TNF-α exposure by altering glutamate balance (72), and high levels of IL-6 can alter neuron excitability, leading to EEG anomalies (73). Moreover, BBB disruption has been associated with increased epilepsy risk (74).

Microglia cells have phagocytic functions and are involved in cognitive functions and neurodegeneration (70, 75). When activated in the case of inflammation, they can secrete IL-1 and TNF-α, further worsening BBB permeability and neuron injury (72). Hence multiple CNS cell types suffer during ICANS, and some also contribute to amplifying the inflammatory state.

##### 2.2.4.5. CNS targeting

CNS infiltration by tumor cells is associated with a dismal prognosis (76), but not all patients with CNS leukemia developed ICANS (21). Moreover, tumor cells expressing the targeted Ag are not required for ICANS development (77), and few patients developing ICANS have CNS involvement. Thus, the presence of direct on-tumor activity of CAR-T cells does not seem to be the driving mechanism of ICANS. A recent study suggests that mural cells of the endothelium in the CNS are CD19 positive and could be targeted by CD19 CAR-T cells in an off-tumor, on-target manner (78). This novel finding could also explain the relatively higher incidence of ICANS with CD19 CAR-T cells compared to other targets. However, CD19 is not the only targeted Ag expressed in the CNS. A single-cell RNA sequencing study confirmed the expression of CD22 in microglia cells (75), which exerts negative regulation of microglia phagocytosis (79). Nevertheless, CAR-T cells targeting CD22 do not seem to lead to a higher incidence of ICANS than other targets (75, 80); therefore, arguing against direct CAR-T cell Ag targets to initiate ICANS. We can hypothesize that targeting mural cells through the CD19 Ag would further intensify vascular leaks and all the consequences cited earlier.

Further analysis and direct comparison of different CAR constructs are still needed to confirm this. On-target, off-tumor toxicity is also suspected for BCMA CAR-T cells, evaluated for patients with multiple myeloma. Non-ICANS neurologic symptoms with parkinsonism have been observed in a subset of patients, and preliminary data seem to indicate BCMA expression in cells located in the basal ganglia (81). Further analyses are needed, but careful neurological follow-up is warranted while using BCMA CAR-T cells.

### 2.3. Management

#### 2.3.1. IL-6 blockade

Tocilizumab is a monoclonal Ab targeting the receptor of IL-6, inducing rapid regression of CRS symptoms and cytokine levels. It is now incorporated in the standard management of CRS (2) and is generally rapidly introduced to better control the development of CRS. Nevertheless, if tocilizumab is potent to limit CRS severity and duration, it does not affect ICANS incidence and severity and is even reported to increase ICANS in some studies (58, 82). One hypothesis to explain the lack of efficacy of IL-6 receptor blockade could be that soluble IL-6 is increased in the serum by blocking the receptor and could cross the altered BBB. In contrast, tocilizumab as an Ab with a higher molecular weight would be limited to blocking the IL-6 receptor in the CNS therefore artificially displacing the detrimental effect on IL-6 from the periphery to the CNS. Because the studies associating tocilizumab with a higher incidence of ICANS had a small sample size and were not randomized, we cannot conclude on the strength of this association, but careful analysis should be prompted for tocilizumab safety use in CRS. One alternative to tocilizumab could be siltuximab as it binds to soluble IL-6 (83) and limits IL-6 increased levels in the CSF (6), but its clinical application would require further studies.

#### 2.3.2. Corticosteroids

Contrasting with IL-6, blockade corticosteroids are recommended in managing ICANS (2) as they will induce rapid and profound systemic anti-inflammatory function by blocking cytokine signaling or adhesion molecules and even induce apoptosis of immune cells (84). However, clinical studies have observed CAR-T cell activity limitation with high-dose corticosteroids in patients with severe ICANS (85–87). More controlled administration of corticosteroids at an earlier setting to limit the length of exposure sometimes showed no detrimental effect on CAR-T cell's efficacy (30, 88, 89). If further studies are needed to describe the potentially detrimental effect of corticosteroids more robustly in CAR-T cells, its use in clinics will not be limited as it is, to date, the most recommended therapy to control ICANS (2). Alternatively, CAR-T cells can be engineered to resist the immunosuppressive effect of corticosteroids. Especially for GBM, where corticosteroids are a cornerstone for managing tumor symptoms, a CAR targeting the IL-13 receptor α2 was further engineered by disrupting the glucocorticoid receptor. Preliminary results seem to show maintained CAR-T cell activity even with a concomitant high dose of dexamethasone (90).

#### 2.3.3. IL-1 blockade

Because IL-1 plays a central role in ICANS, its blockade could offer more precise control of the symptoms and prevent the too-wide anti-inflammatory effects of corticosteroids. Anakinra, an IL-1 receptor antagonist, can penetrate the BBB (56, 91) and is approved for immunological diseases such as Still's disease (92), rheumatoid arthritis (93), and macrophage activation syndrome (94). In recent mouse pre-clinical studies, anakinra better limited neurotoxicity and brain meningeal thickening compared to tocilizumab, without impairing CAR-T cell functionality (25, 31). Many clinical trials are evaluating the potential role of anakinra in limiting the incidence and severity of ICANS (95), and a preliminary study with eight patients observed ICANS control in 50% of them (96).

## 3. Discussion and perspectives

### 3.1. GM-CSF

As myeloid cells, and particularly macrophages, are hypothesized to be the critical cell subtype at the origin of CRS by producing IL-6, blocking their maturation and activation with GM-CSF neutralization have been evaluated. In a mouse model, the use of lenzilumab, an anti-GM-CSF Ab, showed reduced CAR-T cell and myeloid cell infiltration to the CNS without compromising CAR-T cell function and efficacy (40).

### 3.2. IFN-γ

As another approach, inhibition of IFN-γ production with Janus kinase (JAK) inhibitors such as ruxolitinib (97) or itacitinib (98), or with Bruton's tyrosine kinase (BTK) inhibitor ibrutinib (99), all limited CAR-T cell efficacy, even though they induced ICANS remission. A monoclonal IFN-γ blocking Ab emapalumab (100) is available, and may also help to better understand the role of IFN-γ in the development of ICANS.

### 3.3. Adhesion molecules

As VLA4 was described to be overexpressed on CAR-T cells infiltrating the CNS compared to non-CAR-T cells, the blocking Ab natalizumab (101) was evaluated in mice and could prevent CAR-T cell CNS infiltration and reduce inflammation.

### 3.4. CAR constructs

As CAR-T cell proliferation itself is a factor of therapy response but generates cytokine secretion, CAR-T cells are themselves a causative factor for related toxicities. Redesigning CAR constructs by modulating non-signaling domains, including hinge and transmembrane regions or the scFv, may limit the induction of toxicities (102, 103). Editing the scFv for less affinity or with fully human components could retain efficacy while inducing less severe CRS (29, 104). Co-stimulatory domains are also determinant, as CD28 is associated with earlier and potentially more severe CRS than with 4-1BB (30, 105). Nevertheless, reports seem to indicate that JCAR014, a CD19 CAR-T cell with 4-1BB co-stimulatory domain, induces fewer ICANS than other CD19 CAR-T cells (106). It is hypostatized that infusion of equal numbers of CD4 and CD8, which is controlled for JCAR014, could be responsible for this difference, indicating the CD4/CD8 infusion ratio as a determinant factor for ICANS development.

Controlling CAR-T cells already infused in patients through “kill switches” or reversible control of their activity is in development. Adding cell surface proteins such as EGFR or CD20 to target them with cetuximab or rituximab for destruction could be implemented (107), but it would lead to toxicity related to these therapies. Other strategies to embed suicide genes, such as apoptosis inducers with specific triggers toward CAR products, could allow them to induce their self-destruction with a precise signal (108), but CAR constructs are limited in length and may limit the addition of multiple additional systems.

## 4. Conclusion

**Figure 1:**
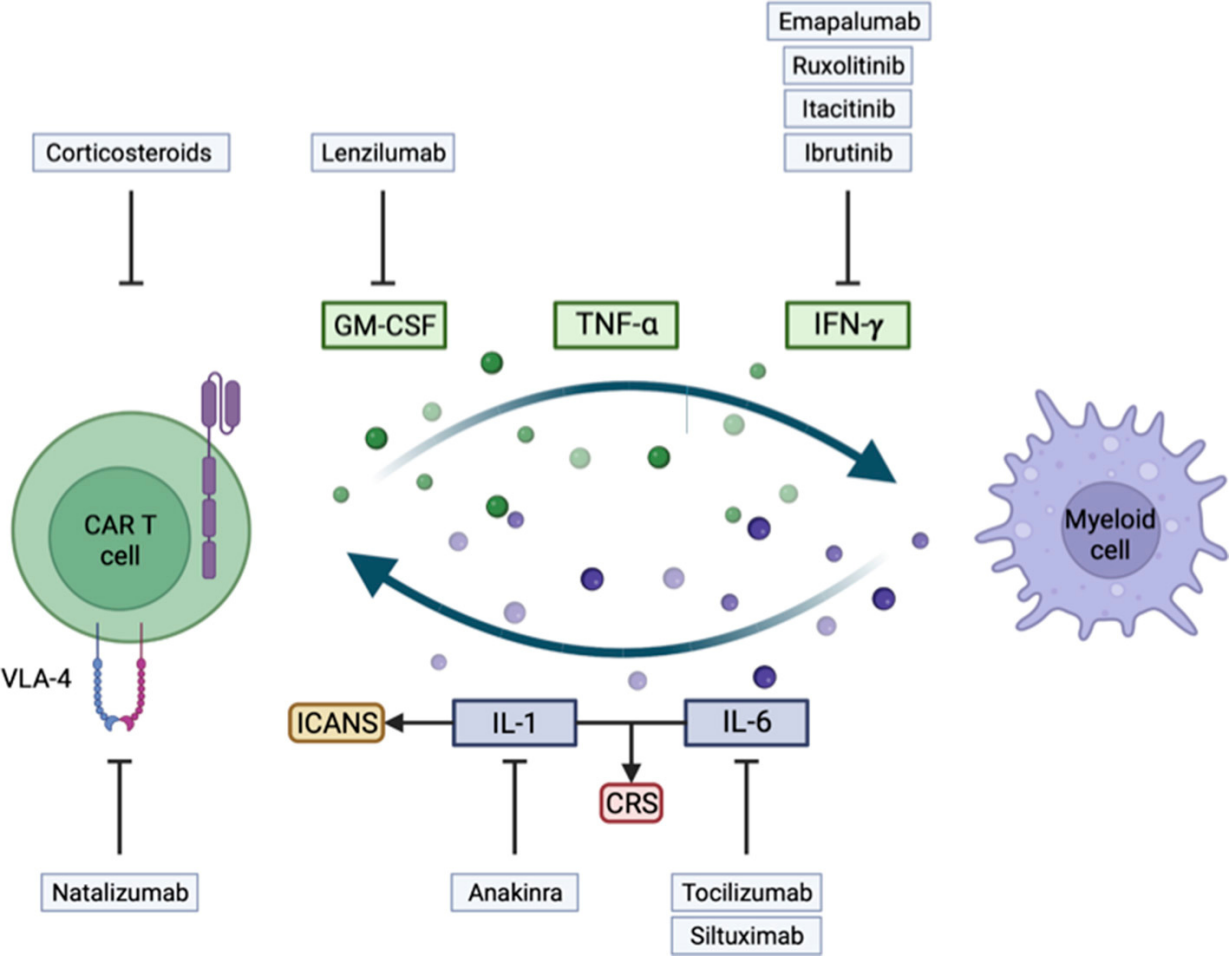
Illustration of key mechanisms involved in the development of immune effector cell-associated neurotoxicity syndrome (ICANS) and cytokine release syndrome (CRS), and their corresponding treatment strategy.

ICANS are now better defined and reported, allowing a real-life grasp of their clinical implication. Improved understanding of mechanisms implicated during ICANS through experimental observations in clinics and with the development of pre-clinical models led to the development of many new strategies to tackle ICANS, as summarized in Table 1 and Figure 1. Nevertheless, some points are not fully understood such as the fact that CAR-T cell studies for solid tumors reported almost no ICANS events in ovary, sarcoma, and glioblastoma trials (7, 8, 109, 110).

**Table 1:** Strategies for ICANS management.

**Molecule**	**Target**	**Efficacy**	**References**
Tocilizumab	IL-6 receptor	Highly proven for CRS but not for ICANS. Sometimes described to increase ICANS incidence	(2, 58, 82)
Corticosteroids	Glucocorticoid receptors	Standard of care for ICANS management	(2, 30, 84–90)
Anakinra	Soluble IL-1	Currently being investigated, more potent for controlling ICANS than IL-6 blockade, as evaluated in many clinical trials	(25, 31, 56, 91–96)
Siltuximab	Soluble IL-6	Currently being investigated, limits IL-6 levels in CSF	(6, 83)
Lenzilumab	GM-CSF	Currently being investigated, limits CAR-T cells and myeloid cells infiltration to the CNS while maintaining CAR-T cell efficacy	(40)
Ruxolitinib	JAK	Currently being investigated, can limit ICANS, but impairs CAR-T cells efficacy	(97)
Itacitinib	JAK	Currently being investigated, can limit ICANS, but impairs CAR-T cells efficacy	(98)
Ibrutinib	BTK	Currently being investigated, can limit ICANS, but impairs CAR-T cells efficacy	(99)
Emapalumab	Soluble IFN-γ	Currently being investigated, could help to study IFN-γ function in ICANS	(100)
Natalizumab	VLA4	Currently being investigated, prevents CAR-T cell CNS infiltration and inflammation	(101)

Better control of CAR-T cell toxicities would probably be the subsequent critical development for broader application, as studies are now evaluating the application of CAR therapies in non-cancerous diseases. We now see development in Ag-specific regulatory T cells to tackle autoimmune diseases (111–114) or CAR-T cells to control organ transplant tolerance (18). In such a situation, on a benefits/risk balance, the weight or risks would have to be well pondered, as high toxicities will be less acceptable.

With broader applications and new CAR subsets coming to the clinic, other toxicities will also be observed and will need further adaptation. We already foresee delayed toxicities with BCMA CAR-T cells inducing parkinsonism-like symptoms in some patients (81), which is not described with the most common CAR-T product to date targeting CD19. CD22 CAR-T cells also seem to drive toxicities at a later time (115) than CD19 CAR-T cells.

Altogether, careful monitoring of patients with current or future CAR-T cell therapies is warranted to allow prompt management and adaptation to unexpected toxicities, with more robust and anticipated management of CAR-T cells toxicities and ICANS in particular, broader application will be facilitated.

## Author contributions

VG and DM wrote, edited, and approved the final version of the manuscript. DM supervised the writing. All authors contributed to the article and approved the submitted version.
